# Case Report: COVID-19–Related Pneumothorax—Case Series Highlighting a Significant Complication

**DOI:** 10.4269/ajtmh.20-0713

**Published:** 2020-07-08

**Authors:** Shaikha D. Al-Shokri, Ashraf O. E. Ahmed, Ahmed Osman Saleh, Mohamed AbouKamar, Khalid Ahmed, Mouhand F. H. Mohamed

**Affiliations:** 1 Internal Medicine Department, Hamad Medical Corporation, Doha, Qatar;; 2 Infectious Disease Department, Hamad Medical Corporation, Doha, Qatar;; 3 Acute Care Surgery Department, Hamad Medical Corporation, Doha, Qatar

## Abstract

COVID-19 is a recent outbreak in China and rapidly spread worldwide. Lung consolidation is the most common radiologic finding of COVID-19 pneumonia. Pneumothorax has been rarely reported as a complication of severe COVID-19 pneumonia. Early recognition and management are detrimental to the outcome. We here report three cases of SARS-CoV-2 infection complicated by pneumothorax. In addition, we present a brief literature review.

## INTRODUCTION

COVID-19 was first reported in Wuhan, Hubei Province, China, in December 2019. The disease is caused by SARS-CoV-2. On January 30, 2020, the WHO declared COVID-19 as a global health emergency.^1,2^

The clinical presentation of SARS-CoV-2 infection varies. Most of the patients develop fever and a flu-like illness.^3^ The diagnosis is confirmed by reverse transcription–PCR (RT-PCR).^3^ Several reports described the radiologic hallmarks of COVID-19, notably ground-glass opacity (GGO) patterns, such as peripheral, nodular, or mass-like GGO.^4^ Furthermore, other features, including bronchiectasis, lymphadenopathy, and pleural and pericardial effusion, have been reported.^5^

Pneumothorax is either uncommon or underreported in patients with COVID-19. Chen et al.^6^ first reported pneumothorax as a rare radiologic feature in 1% of 99 patients, early in the pandemic. The prompt identification and management of pneumothorax are essential. We here report three cases of COVID-19–associated pneumothorax, pneumomediastinum, and subcutaneous emphysema. We further present a relevant literature review.

## CASE PRESENTATIONS

**Figure 1.**
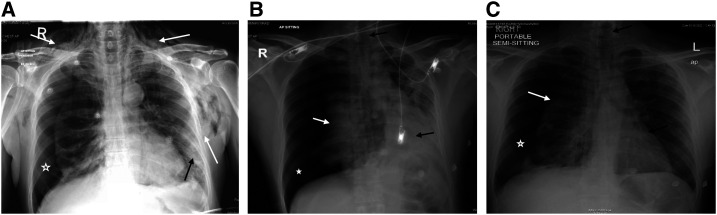
Chest X-ray (CXR) for three patients diagnosed with COVID-19 pneumonia. (**A**) Chest X-ray of case 1 showing bilateral neck and chest surgical subcutaneous emphysema (white arrows). Patchy infiltrates are noted in the left lower lung zone (black arrow). Right pneumothorax (star). (**B**) Chest X-ray of case 2 showing a large right-side tension pneumothorax (star), collapsed right lung (white arrow), deviated mediastinum (black arrows). (**C**) Chest X-ray of case 3 showing a right-sided pneumothorax (star), collapsed right lung (white arrow), and mild mediastinal shift toward the left side (black arrows).

**Figure 2.**
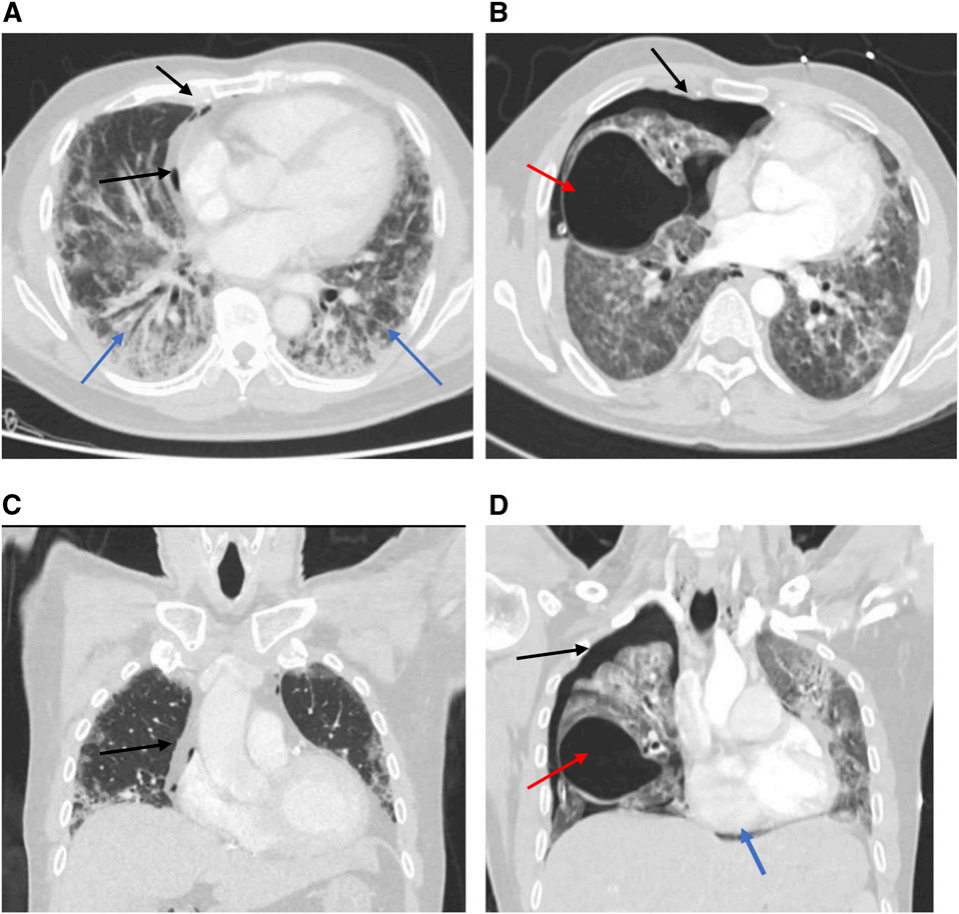
Computed tomography scans of patients diagnosed with COVID-19. (**A** and **B**) Axial and coronal plane of a chest CT scan of case 1 showing bilateral infiltrates (blue arrows) and pneumomediastinum (black arrows). (**C** and **D**) Axial and coronal plane of CT thorax of case 2 showing a moderate right-sided pneumothorax (black arrow) and large air containing bulla right middle lobe (red arrow). The mediastinal structures are shifted to the left with mild pneumomediastinum noted (blue arrow).

The first patient was a 55-year-old man with a history of diabetes who presented with a 1-week history of dyspnea, fever, sore throat, and dry cough. He denied chest pain. On examination, he was febrile, but not tachycardic, nor in distress. His oxygen saturation was 99% in ambient room air. Chest examination and other systemic examinations were unremarkable. Notable laboratory findings included an elevated C-reactive protein (CRP) of 160 mg/L (0–5 mg/L) and serum ferritin of 1,072.0 μg/L (30–553 μg/L) (Table 1). A chest X-ray (CXR) on admission demonstrated infiltrates in the left lower zone. Nasopharyngeal SARS-CoV-2 RT-PCR was positive. The patient received COVID-19 pneumonia treatment (hydroxychloroquine and azithromycin) guided by the local protocol. Two days later, he desaturated and required 5 L of oxygen via a simple mask. A repeat CXR showed a worsening of the bilateral infiltrates. His oxygen requirement was progressively increasing, so he was shifted to noninvasive ventilation. One day later, a repeat CXR showed right-sided pneumothorax with subcutaneous emphysema (Figure 1). A chest tube was inserted. A follow-up chest radiograph showed a resolution of the pneumothorax with residual surgical emphysema. He improved clinically, and the chest tube was removed after 2 days. High-resolution computed tomography (HRCT) was performed to rule out underlying emphysematous bullae or cystic changes. It showed pneumomediastinum with minimal left-sided pneumothorax (Figure 2). The patient was treated conservatively and remained stable. He required an intensive care unit (ICU) admission for 17 days; subsequently, he was transferred to the wards and then discharged in good condition with a follow-up plan.

**Table 1:** Summary of clinical, laboratory, imaging characteristics, and outcomes of three cases with pneumothorax, pneumomediastinum, and subcutaneous emphysema in patients with COVID-19 infection

Patient	Respiratory symptoms, oxygen saturation on presentation	White blood cells (×10^3^/μL)*	C-reactive protein (mg/L)*	Ferritin (μg/L)*	Interleukin-6 level (pg/mL)*	First chest X-ray	High-resolution computed tomography	Required intensive care unit admission	Management	Duration of hospital stay (days)
55-year-old male (patient 1)	Dry cough and sore throat, 99% on ambient air	8.1	160	1,072	Not done	Left lower zone infiltrate	Pneumomediastinum with minimal left-sided pneumothorax	Yes	Chest tube	17
33-year-old male (patient 2)	Fever, dry cough, and dyspnea, 99% on ambient air	9.4	190	750	391	Bilateral ground-glass opacity	Right-side tension pneumothorax, large bulla, and mild pneumomediastinum	Yes	Chest tube	32+
50-year-old male (patient 3)	Fever, dry cough, and dyspnea, 99% on ambient air	10	107	107	79	Bilateral patchy infiltration in all zones	Note done	Yes	Chest tube	10

*Reference values: 4–10 × 10^3^/μL; 0–5 mg/L; ferritin = 30–490 μg/L; lipase = 8–78 U/L; interleukin-6 ≤ 7 pg/mL.

The second patient was a 33-year-old man who presented with a 5-day history of fever and dry cough, and a 3-day history of dyspnea. On admission, his oxygen saturation was 99% on ambient room air. Physical examination was unremarkable. Initial laboratory tests were significant for elevated CRP of 190 mg/L (0–5 mg/L) and ferritin of 750 μg/L (30–553 μg/L) (Table 1). The CXR showed bilateral ground-glass opacity in the middle and lower zones. Nasopharyngeal SARS-CoV-2 RT-PCR test was positive. He was started on COVID-19 treatment as per the local protocol (azithromycin and hydroxychloroquine). His condition worsened, and, on day 3, he was emergently intubated and mechanically ventilated. He was extubated after 3 days. For 2 weeks, he had remained on 4 L oxygen via nasal cannula, maintaining an oxygen saturation of 96%. On day 15, he suddenly deteriorated, and his physical examination revealed absent breath sounds on the right side. A CXR showed large right-sided pneumothorax, which was not present following the extubation (Figure 1). A chest tube was inserted, and the patient’s oxygen saturation improved. High-resolution computed tomography was performed and revealed right-sided tension pneumothorax, large bulla, and mild pneumomediastinum (Figure 2). The chest tube was removed after 10 days. He remained hospitalized and in a good condition at the time of writing this report.

The third patient was a 50-year-old man who presented with a short history of fever, dry cough, headache, and dyspnea. His examinations were unremarkable. The laboratory tests were significant for a rise in the CRP of 107 mg/L (0–5 mg/L) (Table 1). The initial CXR showed diffuse patchy infiltration. SARS-CoV-2 infection was confirmed via the RT-PCR from a nasopharyngeal sample. He received hydroxychloroquine and azithromycin. Seven days later, he complained of shortness of breath and developed hypoxia. A repeat CXR showed right-sided pneumothorax with a mediastinal shift (Figure 1). He was managed with thoracostomy and chest tube. Three days later, the chest tube was removed, and he was completely weaned off oxygen. He was transferred out of the ICU after 10 days to another facility for further observation.

## DISCUSSION

Pneumothorax is air entrapment in the pleural space.^7^ It occurs spontaneously or following trauma. Spontaneous pneumothorax is divided into primary and secondary pneumothorax.^7^

Primary pneumothorax occurs in the absence of underlying parenchymal lung diseases, whereas secondary pneumothorax affects diseased lungs.^8^ Pneumothorax is considered a medical emergency; hence, prompt identification and management are imperative for a favorable outcome.^7,8^

Our patients developed pneumothoraces at different stages of the disease course. The first, second, and third patients developed pneumothorax on days 2, 7, and 15, respectively. Chen et al.^6^ reported pneumothorax as the first presentation in a patient with COVID-19 pneumonia. Subsequent case reports described COVID-19–associated pneumothorax with or without subcutaneous emphysema on the first day of diagnosis.^9–11^ Our case series support the probability that pneumothorax happens in the setting of COVID-19 pneumonia resulting from advanced alveolar damage, and the bronchiolar distortion and narrowing caused by SARS-CoV-2 leading to pulmonary bullae formation. Moreover, the severe cough associated with viral infections increases the intrapulmonary pressure. This may precipitate bullae rupture and pneumothorax formation.^12^

Uniquely, our second patient developed subcutaneous emphysema and pneumomediastinum. Up to our knowledge, this is the second case reporting both findings at the same time. The course of the patients was similar to what is described initially by Wang et al.^13^ as the patient developed this complication later in the course of the disease. This might be due to extensive damage to the lung parenchyma as the disease progresses.

Intubation is known to cause iatrogenic pneumomediastinum, subcutaneous emphysema, and pneumothorax. However, our first and third patients were not intubated, whereas the second patient developed these complications 15 days post-extubation. Nonetheless, we believe intubated patients may have a higher risk of developing these complications, and clinicians should look for these complications whenever sudden deterioration occurs.

At the time of the pandemic with limited patient contact and inadequate physical examination, grave complications can be missed or overlooked, and this may lead to increased morbidity and mortality. Deterioration in the status of SARS-CoV-2–infected patients should not always be attributed to disease progression, and front-liners should keep a broad differential. Two important causes of sudden respiratory compromise specifically related to COVID-19 have been reported. The first is pulmonary embolism, which occurs with a higher prevalence in COVID-19 patients.^14^ The second is pneumothorax, as reported by our case series. Both conditions need prompt diagnosis and intervention. Pneumothorax, being a critical emergency, can easily be diagnosed by physical examination and CXR. Pulmonary embolism can be suspected by looking for signs of deep vein thrombosis in the lower limbs. Point-of-care ultrasound (POCUS) has proven to be effective in COVID-19 diagnosis.^15^ Although the treating clinicians can use POCUS for diagnosis and follow-up, they can still use it to look for signs deep venous thrombosis (veins non-compressibility) and pneumothorax (barcode sign, and absence of lung sliding).^16^ This modality can be useful in resourceful and resource-limited settings.

In conclusion, we reported cases of COVID-19 pneumonia complicated by pneumothorax. In addition, we highlighted the importance of considering this treatable condition as a differential diagnosis besides pulmonary embolism in patients with respiratory compromise. The timely diagnosis and management will reduce COVID-19–associated morbidity and mortality.
